# When husband migrate: effects of international migration of husbands on fetal outcomes, body mass index and gestational weight of female spouses that stay behind

**DOI:** 10.1186/s12889-022-12615-0

**Published:** 2022-02-01

**Authors:** Renuka Jayatissa, Kolitha Wickramage, Buddhini Herath Denuwara, Himali Herath, Ranbanda Jayawardana, Amila Gayan Perera, Nawamali De Alwis

**Affiliations:** 1 Department of Nutrition, Medical Research Institute, P.O. Box 527, Dr. Danister De Silva Mawatha, Colombo 08, 0080 Sri Lanka; 2 Global Migration Health Research and Epidemiology Unit, Migration Health Division, Paseo De Roxas Makati City, 1226 Manila, Philippines

**Keywords:** Pre-pregnancy, BMI, Gestational weight gain, Migrant husband, Lactating women, Low birthweight

## Abstract

**Background:**

International labour migration continues to be an integral component in Sri Lanka’s economic development. Previous research indicates an adverse perinatal outcome in association with low maternal pre-pregnancy body mass index (PBMI) and gestational weight gain (GWG). However, evidence of this association is limited in migrant families. This study aims to investigate the associations between PBMI, GWG among lactating mothers (LM), and fetal outcomes in migrant households, where the father is the migrant worker.

**Methods:**

A secondary data analysis was done using a nationally representative sample of 7,199 LM. There were 284 LM whose husbands were international migrant workers. Maternal factors were taken as PBMI<18.5 kg/m^2^ and GWG<7kg. Preterm birth and low birth weight (LBW) were taken as fetal outcomes. Binary logistic regression was performed to assess the associated factors.

**Results:**

There was significant difference between LM from migrant and non–migrant households with regards to place of residency, ethnicity, household monthly income, household food security, average household members, husband’s education and husband’s age. Among migrant, PBMI<18.5 kg/m^2^ was associated with current BMI and mode of delivery. Migrant LM had significantly higher weight gain (≥12 kg) during pregnancy (*p*=0.005), were multiparous (*p*=0.008), delivered in private hospital (*p*=0.000), lesser percentage of underweight (*p*=0.002) and higher birthweight (*p*=0.03) than non-migrant LM. Logistic regression model revealed that for each kilogram increment in birthweight and GWG, preterm delivery decreased by 89%(OR=0.11;95%CI:0.04-0.28) and LBW decreased by 12%(OR=0.89;95%CI:0.81-0.97) respectively. Caesarean deliveries were positively associated with low GWG.

**Conclusion:**

Our study showed LM in migrant families had invested remittances to utilize private health facilities for deliveries, to improve weight gain during pregnancy and adequate PBMI to deliver higher birth weight babies. In depth study is needed to understand further utilisation of remittances to improve fetal outcomes by increasing birthweight and GWG in migrant families.

## Background

Migration for purposes of work and employment (economic migration) is the most predominant form of international migration globally [1]. Labour migrants comprise nearly two-thirds of the 281 million international migrant population [2]. Sri Lanka’s International Migrant Workers (IMW) are a vital part of the economy, with over 200,000 Sri Lankans emigrating for work annually [3]. Once a highly feminized labour force, the most recent foreign data show 60.1% of Sri Lanka’s foreign employment workforce are males [3]. Most Sri Lanka’s migrant workers are young people of reproductive age with 81% below 49 years of age, with half in the 20-34 years age group [4]. Majority of Sri Lankans are employed abroad as domestic maids or labourers [3, 5]. Despite the monetary benefits to migrants and their families through inbound remittance flows, the health outcomes for IMWs and their left-behind families show a mixed patterns of health vulnerabilities [6–12]. In June 2013, the Sri Lanka Bureau of Foreign Employment (SLBFE) introduced a ‘Family Background Report’ (FBR) regulation, banning prospective women domestic workers with children under the age of five years from migrating for work overseas [13]. The policy was intended to decrease departures of lower-skilled female migrant worker groups (such as domestic maids) to limit cases of abuse [1] and safeguard the rights of ‘left-behind’ children [11]. Since the FBR policy inception, published studies that have explored the health impact of lactating mothers on migrating male spouses; are not available in Sri Lanka, despite the rapid increase in outbound male migration.

In the review of literature, we found only one study from Mexico that examined pregnancy outcomes of women in labour migrant families [14]. It was found that international migration had a positively significant effect on perinatal outcomes of women in both countries of origin and in countries of destination, with reduced risk of Low Birth Weight (LBW) in women in migrant households [14].

Previous literature has revealed that women with low Gestational Weight Gain (GWG) have a higher risk of LBW [15–21]. Weight gain in the second half (after 20 weeks) of pregnancy has a more pronounced effect on the growth and the birth weight of the baby. Poor weight gain especially in the third trimester is associated with LBW, which is associated with a higher incidence of infant mortality and morbidity, poor cognitive development and learning disability. They are also prone to have non communicable diseases like heart disease, hypertension and diabetes mellitus in later life [22]. Therefore, maintaining a proper GWG is important to have a baby with good birth weight.

Multiple studies have revealed GWG to be related to the risk of pregnancy complications; such as higher risk of gestational diabetes, pre-eclampsia, pregnancy induced hypertension, preterm delivery and large for-gestational age (LGA) births, small for gestational age (SGA) births, neonatal seizures, low Apgar score, neonatal intensive care unit admission, and infant death. GWG is attributed as a modifiable risk factor for adverse prenatal outcomes [15–42]. Despite large volume flows of international labour migrants from many low to middle-income countries, studies in published literature could not be found regarding pregnant and lactating women of migrant husbands.

In Sri Lanka, there is a persistent high prevalence of LBW [43, 44]. The preterm birth was 9.8% [43], low pre pregnancy BMI was 11.2% and mean GWG was 9.4±5 kg [43]. However, there has been limited research on the topic of migration and preterm births, LBW, GWG and pre pregnant BMI (PBMI).

Hence the aim of this study was to investigate the effect of international migration of husbands on maternal factors (PBMI and GWG), and fetal outcomes (preterm delivery and LBW) of female spouses that stay behind.

## Methods

Migrant households were defined as those in which husband of the lactating women migrated internationally for labour at the time of study, otherwise the household was considered as non-migrant.

### Data source

Data of the Sri Lanka national nutrition and micronutrient study of lactating women were used for analysis. Data was collected during May to November 2015 [43]. This was a stratified, multi-stage cluster study carried out in all 25 districts in Sri Lanka, each district was treated as separate strata. Altogether 750 clusters (public health midwife areas) were selected, 30 from each district. Public Health Midwife (PHM) is the lowest level of health care officer provide services for about 3000 population and PHM maintains birth and immunization register for respective population under care. Second stage sampling, 10 lactating mothers were randomly selected using computer generated random numbers from the birth and immunization register, which is maintained by the PHM for respective population under care [22]. In the original study, lactating mother was defined as women delivered the baby within last 6 months. Women with and women with psychiatric illnesses, cognitive impairment and mentally subnormal were excluded. A total of 7199 LM completed interviewer administered questionnaire at household level. The key advantage of using the dataset was data collected and measurements were done by the trained research staff in the department of nutrition, Medical Research Institute [43]. The study was conducted in accordance with the Declaration of Helsinki, and the protocol was approved by the Ethics Committee of Medical Research Institute, Colombo.

### Measures of maternal factors and fetal outcomes

**Fig. 1:**
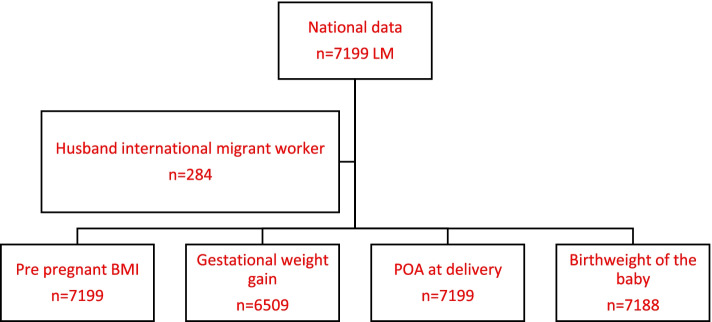
Flow diagram of the sample

Based on literature, PBMI and GWG was selected as maternal factors. None of the study participants in the migrant sample, had gestational diabetes or pregnancy-induced hypertension. BMI at the first clinic visit during first trimester was considered as PBMI. Weight at first clinic visit during first trimester and weight at last clinic visit during third trimester was used to calculate GWG. BMI<18.5 kg/m^2^ (underweight) and GWG<7.0 kg (below 25^th^ percentile of median GWG) was taken as maternal factors. Preterm delivery (delivered at Period of Amenorrhea [POA] <37weeks) and LBW (birthweight <2.5kg) was taken as fetal outcomes. Figure 1 shows the flow diagram of the sample.

In the original study [43], pregnancy related data were extracted from the pregnancy records, which was maintained by the PHM and attending Obstetrician or doctor [22].

### Data analysis

Current BMI (BMI at the interview) was calculated by dividing the weight in kilograms by squared height in meters. Both current BMI and PBMI was categorised into underweight (<18.5); adequate (18.5-24.9); overweight (25.0-29.9) and obesity (≥ 30) kg/m^2^, according to World Health Organisation (WHO) guidelines [22]. Descriptive analysis by migrant and non-migrant households was examined.

Remittances were included when estimating total household income. Total household income was categorised into low income (<35,000 LKR) and high income (≥35,000 LKR) considering the mean household income of the households. Household food insecurity in the dataset was measured using the World Food Programme criteria [43]. It included a household food consumption adequacy score that was based on food groups consumed one week prior to the study, estimating the expenditure on food as a percentage of the total household expenditure, then categorisng the households into 3 groups indicating different levels of food access. Finally, three levels of food insecurity were deliberated as severe, moderate and food secure.

Explanatory variables (covariates) used in the analysis included: place of residency (urban/rural), number of household members, household food insecurity (severe, moderate and food secure), total household monthly income (<35,000 LKR/≥35,000 LKR), ethnicity (Muslim and non-Muslim). Maternal variables included age, years of schooling, current BMI, parity (primi/multi), place of delivery (state/private hospital), type of delivery (vaginal/caesarean, forceps and others), and husbands’ age and years of schooling. Household food insecurity and monthly household income were considered as possible confounders.

Normality of the data was checked, and normally distributed data was presented as mean (SD) and analyzed using chi-square and ANOVA. Aim of this study was to estimate in migrant families, the probability of being underweight during pre-pregnant period (BMI<18.5kg/m^2^), low weight gain in pregnancy (<7kg), preterm delivery (POA<37weeks) and delivering LBW baby (birthweight <2.5kg) to changes in the explanatory variables in migrant families. The percentage of missing values across the four variables varied between 0 to 9.6% due to incomplete data in the original dataset. Gestational weight gain had comparatively more missing data due to absence of weight at last clinic visit. The analysis was restricted to the complete cases only.

Binary logistic regression model was used to examine the risk of LBW, preterm delivery, PBMI and GWG as binary dependent variables. PBMI and GWG was grouped as BMI<18.5kg/m^2^=1 / BMI≥18.5kg/m^2^=0 and GWG<7kg=1 / GMG≥7kg=0 respectively. Preterm delivery and LBW was grouped as POA <37weeks=1 / ≥37weeks=0 and birth weight <2.5kg=1 / ≥2.5kg=0 respectively. Rural residency, severe household food insecurity, primi parity, delivered in the private hospital, caessarian/vacuum/forceps delivery, Muslim ethnicity, years of schooling of LM and husbands’ 1-10 years, were considered as a value of 1. The significant covariates were used for each model. Overall goodness-of-fit was assessed through Hosmer-Lemeshow test, likelihood ratio test and Nagelkerke R^2^. All statistical analyses were conducted using SPSS Statistical Software version 20. Statistical significance was considered at *p*<0.05.

## Results

A total sample size of 7199 was included in our study after excluding missing information. Husbands of 284 lactating mothers were international migrant workers, who were coded as ‘migrants’ and the rest as ‘non-migrant’.

As shown in Table 1, the migrant sample had significantly higher percentage from, urban sector (19.4 vs 11.2%; *p*<0.001), Muslim ethnicities (38 vs 10.9%; *p*<0.001), household income of ≥Rs.35,000 (57.0 vs 34.4%; *p*<0.001) and food secure households (72.5 vs 56.3%; *p*<0.001). Mean age of husbands (33.2 vs 32.3 years; *p*<0.05), mean years of husband’s education (11 vs 10.5 years; *p*<0.001) and mean household members (5.4 vs 4.9; *p*<0.001) was significantly higher in the migrant than in the non-migrant.

**Table 1:** Basic characteristics of households and maternal factors in migrant and non-migrant participants

	Migrant	Non-migrant	Total
**Household characteristic**	**No. (%)**		
Place of residency***			
Urban	55 (19.4)	775 (11.2)	830 (11.5)
Rural	229 (80.6)	6140 (88.8)	6369 (88.5)
Ethnicity***			
Sinhala	96 (33.8)	4335 (62.7)	4431 (61.6)
Tamil	80 (28.2)	1823 (26.4)	1903 (26.4)
Muslim	108 (38.0)	757 (10.9)	865 (12.0)
Household income (Rs.) ***			
< 35000	122 (43.0)	4538 (65.6)	4660 (64.7)
>35000	162 (57.0)	2377 (34.4)	2539 (35.3)
Household food security***			
Severe food insecure	2 (0.7)	312 (4.5)	314 (4.4)
Moderate food insecure	76 (26.8)	2709 (39.2)	2785 (38.7)
Food secure	206 (72.5)	3894 (56.3)	4100 (57.0)
	**Mean (SD)**		
Number of household members***	5.4 (1.8)	4.9 (1.4)	4.9 (1.4)
Husband’s age in years*	33.2 (5.3)	32.3 (5.9)	32.4 (5.9)
Husband’s years of schooling^***^	11 (1.6)	10.5 (1.9)	10.5 (1.9)
	**Mean (SD)**		
Mother’s age in years	29.2 (5.3)	28.8 (5.6)	28.8 (5.6)
Mother’s years of schooling	11.0 (1.8)	10.8 (1.8)	10.8 (1.8)
Current BMI (kg/m^2^)	23.8 (4.2)	23.3 (4.2)	23.4 (4.2)
Pre-pregnant BMI (kg/m^2^)	22.5 (4.3)	22.0(4.4)	22.0 (4.4)
Weight gain during pregnancy (kg)***	10.2 (1.8)	9.3 (4.9)	9.3 (4.9)
	**No. (%)**		
Parity^**^			
Primipara (1)	111 (39.1)	2210 (32.0)	2321 (32.2)
Multipara (>1)	173 (60.9)	4705 (68.0)	4878 (67.8)
Place of delivery***			
Government hospital	261 (91.9)	6739 (97.5)	7000 (97.2)
Private hospital	23 (8.1)	176 (2.5)	199 (2.8)
**Type of delivery**			
Normal vaginal delivery	174 (61.3)	4583 (66.3)	4757 (66.1)
Caessarian section/forceps/vacuum	110 (38.7)	2332 (33.7)	2442 (33.9)
Prepregnant BMI groups**			
Underweight (<18.5)	40 (15.6)	1437 (22.9)	1477 (22.6)
Adequate (18.5-24.9)	159 (61.9)	3324 (53.0)	3488 (53.3)
Overweight (25.0-29.9)	39 (15.2)	1202 (19.2)	1241 (19.0)
Obese (>=30.0)	19 (7.4)	313 (5.0)	332 (5.1)
Weight gain during pregnancy (kg)**			
< 7.0	47 (18.5)	1577 (25.2)	1624 (25.0)
7.5-11.9	107 (42.1)	2238 (45.4)	2945 (45.2)
≥ 12.0	100 (39.4)	1840 (29.4)	1940 (29.8)
**n**	**284**	**6915**	**7199**

^*^ *p*<0.05

^**^ *p*<0.01

^***^ *p*<0.001

There were no significant difference between age of the LM, years of schooling, current BMI and type of delivery between migrant and non-migrants. However, the migrant sample had significantly higher percentage of primiparous LM (39.1 vs 32.0%; *p*<0.01), more LM delivered in private hospital (8.1 vs 2.5%; *p*<0.001), less underweight LM (15.6 vs 22.9%; *p*<0.01) and high weight gain (≥12kg) during pregnancy (37.7 vs 28.5%; *p*<0.01) than the non-migrant.

The relationship between fetal outcomes between migrants and non-migrants is shown in Table 2. There is a significantly higher proportions of ≥3.5 kg birth weight babies delivered by migrant than non-migrant LM (14.8 Vs 10.0%; *p*<0.05).

**Table 2:** Fetal outcomes of lactating mothers in migrant vs non-migrant participants

Characteristics	Migrant	Non-Migrant	Total
		Number (%)	
**Period of gestation at delivery**			
Preterm delivery (< 37 weeks)	32 (11.3)	675 (9.8)	707 (9.8)
Term delivery (> 37 weeks)	252 (88.7)	6240 (90.2)	6492 (90.2)
Birth weight of the baby (kg)*			
<2.5	47 (16.5)	1105 (16.0)	1152 (16.0)
2.5 – 3.4	195 (68.7)	5104 (73.9)	5299 (73.7)
≥ 3.5	42 (14.8)	693 (10.0)	735 (10.2)
	**Mean (SD)**		
Period of gestation at delivery (weeks)	38.4 (1.8)	38.42 (1.72)	38.42 (1.72)
Birth weight of the baby (kg)	3.0 (0.5)	2.9 (0.5)	2.9 (0.5)
n	**284**	**6915**	**7199**

^*^ *p*<0.05

The binary logistic model applied for maternal factors of PBMI<18.5 kg/m^2^, GWG<7.0kg and fetal outcomes of migrant LMs is depicted in Table 3. Logistic model revealed that among migrant LM, BMI at the time of interview and caesarian delivery was negatively associated with underweight (BMI<18.5 kg/m^2^) LM during pre-pregnancy. Living in rural areas and primi parity were negatively associated and caesarian deliveries were positively associated with GWG<7.0 kg. One kilogram increased in birth weight reduced preterm deliveries by 89%. In addition, one kilogram increase in GWG reduced low birth weight by 12%.

**Table 3:** Factors associated with prepregnant BMI, weight gain, preterm deliver and LBW in binary logistic regression model

Independent variables	B	SE	P	Exp(B)	95% CI for Exp(B)	
					Lower	Higher
**Dependent variable = Pre pregnant BMI <18.5**						
Age of LM	-.004	.070	.949	.996	.868	1.142
Age of husband	.102	.070	.150	1.107	.964	1.271
Current BMI	-.772	.123	.000	.462	.363	.589
Parity=Primi	.889	.532	.095	2.433	.857	6.908
Delivery= Caesarean/forceps/vacuum	-1.559	.608	.010	.210	.064	.692
Constant	11.398	2.541	.000			
Log likelihood	122.327					
N observation	284					
Model χ2=108.567; df=5; *p*<0.001; Hosmer-Lemeshow test=0.144; Nagelkerke R^2^=0.571						
**Dependent variable = Weight gain < 7.0 kg**						
Sector=Rural	-.840	.333	.012	.432	.225	.829
Place of delivery=private hospital	-.645	.558	.248	.525	.176	1.568
Husband’s education=1-10 years	.027	.399	.945	1.028	.470	2.246
Delivery=Caesarean/forceps/vacuum	.653	.289	.024	1.922	1.091	3.385
Parity=Primi	-.832	.314	.008	.435	.235	.805
LM education=1-10 years	.332	.383	.386	1.393	.658	2.952
Constant	1.670	.676	.014			
Log likelihood	308.625					
N observation	263					
Model χ^2^=54.886; df=1; *p*<0.001; Hosmer-Lemeshow test=0.461; Nagelkerke R^2^=0.114						
**Dependent variable = Preterm delivery (POA <37 wks)**						
Weight gain	-.001	.041	.979	.999	.922	1.082
Birth weight	-2.230	.487	.000	.108	.041	.279
Constant	4.221	1.270	.001			
Log likelihood	168.385					
N observation	284					
Model χ^2^=110.895; df=1; *p*<0.001; Hosmer-Lemeshow test=0.742; Nagelkerke R^2^=0.185						
**Dependent variable = LBW (Birth weight<2.5kg)**						
Weight gain kg	-.123	.048	.011	.885	.805	.972
Preterm delivery=Yes	-.330	.368	.370	.719	.350	1.479
Place of residency=Rural	-1.469	.453	.001	.230	.095	.560
Delivery= Caesarean/forceps/vacuum	.646	.425	.129	1.907	.829	4.386
Age of LM	-.038	.051	.458	.963	.871	1.064
Age of husband	-.026	.051	.620	.975	.881	1.078
Current BMI	.019	.122	.874	1.020	.802	1.296
Pre pregnant BMI	-.100	.122	.411	.905	.713	1.148
Constant	4.567	1.679	.007			
Log likelihood	211.288					
N observation	263					
Model χ^2^=92.861; df=1; *p*<0.001; Hosmer-Lemeshow test=0.611; Nagelkerke R^2^=0.177						

## Discussion

This study is the first to explore the effect of international migration on pre-pregnancy body mass index, gestational weight gain, and fetal outcomes of women who stay behind, utilising data from a national survey in Sri Lanka. In this sample, there is a significant difference between characteristics of households and husbands of migrant and non–migrant. These findings are not compatible with previous study conducted in Sri Lanka among children under five years left behind by migrant parents highlighting that the migrant population in this study is different from the general population [45]. However, there is no significant difference of baseline characteristics of LM between migrant and non-migrant in relation to mean age, mean years of schooling, mean current BMI and mean PBMI. Labour migration has a high degree of heterogeneity with employment in skilled, low-skilled or regular occupations and through undocumented flows. It is worthwhile to investigate further into the migrant typology.

Our study showed that migrant LM, had a significantly higher GWG (*p*=0.005), delivered in private hospital (*p*=0.000), had a lesser percentage of those who were underweight (*p*=0.002) and a higher birth weight (*p*=0.03) than non-migrant LM. It indirectly indicates that remittances have been utilized to obtain private facilities, to improve weight gain, to better feed during pre-pregnant period and to deliver higher birth weight babies.

As a low middle-income country, Sri Lanka is still fighting with maternal under nutrition and persistently high prevalence of low birthweight babies [43]. Regression analysis revealed that for each kilogram increment in birthweight, preterm delivery decreased by 89% (OR=0.11;95% CI 0.04-0.28). Furthermore, with each kiligram increment in GWG, deliver of LBW babies decreased by 12% (OR=0.89;95% CI 0.81-0.97). Low birth weight is associated with a higher incidence of infant mortality and morbidity, poor cognitive development, learning disability and including a tendency to develop non communicable diseases such as heart disease, hypertension and diabetes mellitus in later life [22]. Hence this finding will help to improve gestational weight gain in migrant LM who invest the remittances on good birth weight of their babies [46].

Our study finding are in line with many studies [15–21], as they confirm poor maternal weight gain as an an important risk factor for LBW. Even though not focused on migrant households, a study conducted among Vietnamese women (*n* = 228) in 2019 revealed that gestational weight gain was positively associated with birth weight and birth weight-for-age z-score (all *p* ≤ 0.006) [15]. Another study conducted in China (*n*=3172) disclosed that inadequate GWG was a risk factor for low birth weight (OR=1.7; CI=1.08–2.6; *p*< 0.05) [19]. A study conducted in Germany (*n* = 200) in 2016 revealed that each kilogram of weight gained during pregnancy leads to an increase in birth weight by 20 grams (95 % CI 3–36) [18].

The logistic model revealed that among migrant LM, BMI at the time of interview and caesarian delivery were negatively associated with underweight (BMI<18.5) LM during pre-pregnancy. Caesarian deliveries were positively associated with GWG<7.0 kg. Within 25 cohort studies from Europe and North America, internal migrant LM revealed that pre-pregnancy weight and the magnitude of gestational weight gain were associated with risk for any adverse outcome such as cesarean delivery [29]. Data is scarce regarding women of migrant husband.

The strength of this study is that, the sample is obtained from a nationally representative study in Sri Lanka. The limitations in this study include a small sample size of migrant LM, which needs to be explored further. The data of pre-pregnancy BMI and GWG were obtained and calculated based on pregnancy records, which may lead to dilemmas in validity. There were missing data regarding birth weight and GWG and the data analysis was conducted for available data.

## Conclusions

It appears that our study sample had invested remittances to utilize private health facilities for deliveries, to improve weight gain during pregnancy and prepregnant BMI to deliver higher birth weight babies. In depth studies are needed to unburden these associations considering the length of migration, cycles of repeat migration, type of overseas employment and remittance levels. There is a need to build capacities of migrant families in better utilizing and investing remittances for better fetal outcome.

## Data Availability

The data sets generated and analyzed during the current study are not publicly available due to not obtaining ethical clearance to share data publicly but are available from the corresponding author on reasonable request.

## Abbreviations

BMI: Body Mass Index
95 % CI: 95 % confidence interval
DF: Degree of Freedom
GWG: Gestational weight gain
IMW: International Migrant Workers
IQR: Inter Quartile Range
LBW: Low birth weight
LGA: Large for-gestational age
LM: Lactating mother
MD: Mean Difference
NCD: Non-Communicable Diseases
PHM: Public Health Midwife
POA: Period Of Gestation
SD: Standard deviation
SGA: Small for gestational age
SLBFE: Sri Lanka Bureau of Foreign Employment
WHO: World Health Organisation
